# A stitch in time: Efficient computation of genomic DNA melting bubbles

**DOI:** 10.1186/1748-7188-3-10

**Published:** 2008-07-17

**Authors:** Eivind Tøstesen

**Affiliations:** 1 Department of Tumor Biology, Norwegian Radium Hospital, N-0310, Oslo, Norway; 2 Department of Mathematics, University of Oslo, N-0316, Oslo, Norway

## Abstract

**Background:**

It is of biological interest to make genome-wide predictions of the locations of DNA melting bubbles using statistical mechanics models. Computationally, this poses the challenge that a generic search through all combinations of bubble starts and ends is quadratic.

**Results:**

An efficient algorithm is described, which shows that the time complexity of the task is O(NlogN) rather than quadratic. The algorithm exploits that bubble lengths may be limited, but without a prior assumption of a maximal bubble length. No approximations, such as windowing, have been introduced to reduce the time complexity. More than just finding the bubbles, the algorithm produces a stitch profile, which is a probabilistic graphical model of bubbles and helical regions. The algorithm applies a probability peak finding method based on a hierarchical analysis of the energy barriers in the Poland-Scheraga model.

**Conclusion:**

Exact and fast computation of genomic stitch profiles is thus feasible. Sequences of several megabases have been computed, only limited by computer memory. Possible applications are the genome-wide comparisons of bubbles with promotors, TSS, viral integration sites, and other melting-related regions.

## Background

Models of DNA melting make it possible to compute what regions that are single-stranded (ss) and what regions that are double-stranded (ds). Based on statistical mechanics, such model predictions are probabilistic by nature. Bubbles or single-stranded regions play an essential role in fundamental biological processes, such as transcription, replication, viral integration, repair, recombination, and in determining chromatin structure [1,2]. It is therefore interesting to apply DNA melting models to genomic DNA sequences, although the available models so far are limited to *in vitro *knowledge. Genomic applications began around 1980 [3,4], and have been gaining momentum over the years with the increasing availability of sequences, faster computers, and model development. It has been found that predicted ds/ss boundaries often are located at or very close to exon-intron junctions, the correspondence being stronger in some genomes than others [5-9], which suggested a gene finding method [10]. In the same vein, comparisons of actin cDNA melting maps in animals, plants, and fungi suggested that intron insertion could have target the sites of such melting fork junctions in ancient genes [11,12]. In other studies, bubbles in promotor regions were computed to test the hypothesis that the stability of the double helix contributes to transcriptional regulation [13-18]. The role of TATA bubbles and their lifetimes has been further discussed using a stochastic model of dynamics based on single molecule experiments [19,20]. Bubbles induced by superhelicity have also been found to correlate with replication origins as well as promotors [21-24]. In addition to the testing of specific hypotheses, a strategy has been to provide whole genomes with annotations of their melting properties [25,26]. Combined with all other existing annotations, such melting data allow exploratory data mining and possibly to form new hypotheses [27]. For example, the human genomic melting map was made available, compared to a wide range of other annotations, and was shown to provide more information than the local GC content [26].

In the genomic studies, various melting features have proved to be of particular interest. These include the bubbles and helical regions, bubble nucleation sites, cooperative melting domains, melting fork junctions, breathers, sites of high or low stability, and SIDD sites. Most often we want to know their locations, but additional information is sometimes useful, such as probabilities, dynamics, stabilities, and context. DNA melting models based on statistical mechanics are powerful tools for calculating such properties, especially those models that can be solved by dynamical programming in polynomial time. For many features of interest, however, algorithms remain to be developed to do such predictions. The existing melting algorithms typically produce melting profiles of some numerical quantity for each sequence position. The prototypical example is Poland's probability profile [28], but also profiles of melting temperatures (melting maps), free energies or other quantities are computed per basepair. The result can be plotted as a curve, while the wanted features often have the format of regions, junctions and other sites. Some genomics data mining tools also require data in these formats rather than curves. As a remedy, melting profiles have been subjected to *ad hoc *post-processing methods to extract the wanted features, such as segmentation algorithms [26], thresholding [25], and relying on the eye through visualization [9,12].

**Figure 1:**
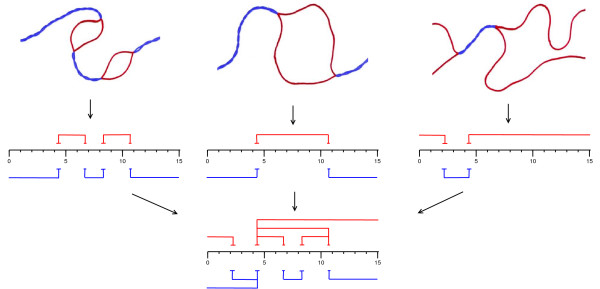
**What is a stitch profile diagram?**. At the top are sketched three alternative DNA conformations at the same temperature. In the middle diagrams, the sequence location of each helical region (blue) and each bubble or single-stranded region (red) is represented by a stitch. At the bottom, the three "rows of stitches" are merged into a stitch profile diagram.

In previous work, we developed an algorithm that identifies regions of four types: helical regions, bubbles (internal loops), and unzipped 5' and 3' end regions (tails) [29-31]. The algorithm produces a *stitch profile*, which is a probabilistic graphical model of DNA's conformational space. A stitch profile contains a set of regions of the four types. Each region is called a *stitch*, because of the way they can be connected in paths. The stitch profile algorithm computes the location (start and end) of each stitch and the probability of that region being in the corresponding state (ds or ss) at the specified temperature. A stitch profile can be plotted in a *stitch profile diagram*, as illustrated in Figure 1. The location of a bubble or helix stitch is not given as a precise coordinate pair (*x*, *y*), but rather as a pair of ds/ss boundaries with fuzzy locations. For each ds/ss boundary, the range of thermal fluctuations is computed and given as an interval. A stitch profile indicates a number of alternative configurations, both optimal and suboptimal, as illustrated in Figure 1. In contrast, a melting map would indicate the single configuration at each temperature, in which each basepair is in its most probable state.

A stitch profile thus provides some features, e.g. bubbles, that would be of interest in genomic analyses. However, the previously described algorithm for computing stitch profiles [29] has time complexity *O*(*N*^2^). Genomics studies often require faster algorithms, both to compute long sequences and to compute many sequences. In this paper, therefore, an efficient stitch profile algorithm with time complexity *O*(*N *log *N*) is described, and the prospects of computing genomic stitch profiles are discussed. The original algorithm [29] is referred to as Algorithm 1, while the new algorithm is referred to as Algorithm 2.

The reduction in time complexity has been achieved without introducing any approximation or simplification such as windowing. The usual tradeoff between speed and precision is therefore not involved here. The output of Algorithm 2 is not of a lower quality, but identical to Algorithm 1's output. Algorithm 1 was simply inefficient. However, it was not obvious that this problem has time complexity *O*(*N *log *N*), which is the same as computing melting profiles with the Poland-Fixman-Freire algorithm [32]. It would appear that the stitch profile had greater complexity, for example, that the search for all bubble starts and ends would be quadratic. On the other hand, we know that bubbles may be small compared to the sequence length. Algorithm 2 detects such circumstances in an adaptive way, without assuming a maximal bubble length.

## Methods

The proper way of computing DNA conformations, as well as other macromolecular structures, is to consider a *rugged landscape *[33,34]. As an abstract mathematical function, a landscape applies to widely different complex systems, for example, fitness landscapes in evolutionary biology for defining populations and species. The ruggedness implies many local maxima and minima on many levels. In optimization, the task would be to avoid all the "false" local optima and find the global optimum. That is not what we want. On the contrary, we would prefer to include most of them.

A local optimum corresponds to an instantaneous conformation or *microstate *that is more fit or stable than its immediate neighbors. However, fluctuations over time cover a larger area in the landscape around the local optimum, which is defined as a *macrostate*. A macrostate can not simply be associated with a local optimum, because it usually covers many local optima. On the other hand, a local optimum may be part of different macrostates. Fluctuations are biologically important, as they represent stability and robustness, rather than noise and uncertainty [35]. Conformations are properly represented by macrostates, not microstates. We want to characterize the whole landscape of DNA conformations by a set of macrostates. 

More specifically, this article considers certain probability landscapes, in which the probability peaks are the macrostates. The algorithmic task is to find a set of peaks. Automatic peak detecting is applied in various kinds of spectroscopy (NMR), spectrometry (mass-spec), and image segmentation (e.g. in astronomy), but these algorithms usually do not consider any hierarchical aspects. Hierarchical peak finding is analogous to hierarchical clustering, which is widely used in bioinformatics. However, our approach is closely related to the hierarchical analyses of energy landscapes and their barriers in studies of dynamics, metastability, and timescales [36-39]. The algorithm uses a subroutine for finding hierarchical probability peaks in one dimension, described in the next section.

### 1D peaks

This section briefly revisits the 1D peak finding method and the use of a nonstandard pedigree terminology [29]. Here is a generic formulation of the problem: Let *p*(*x*) be some probabilities (possibly marginal) defined for *x *= 1, ..., *N *. What are the peaks in *p*(*x*)? The computational task is divided into two steps. The first step is to construct a discrete tree of possible peaks, and the second step is to select peaks by searching the tree.

**Figure 2:**
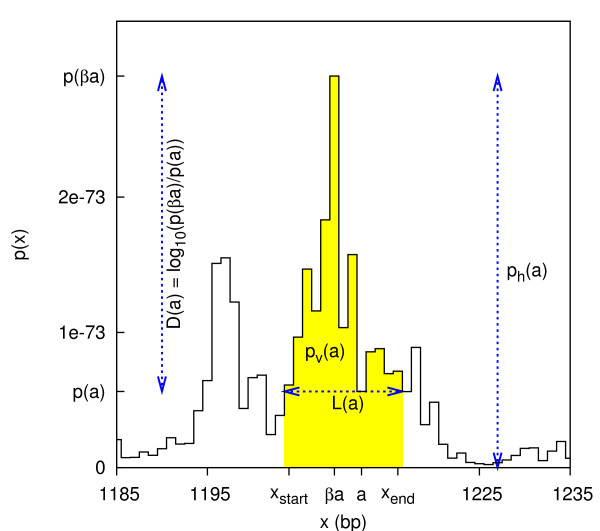
**Example of a 1D peak**. This peak in *p*(*x*) has peak volume (yellow area) *p*_v_(*a*) = 1.5 × 10^-72^, while the peak height is *p*_h_(*a*) = 2.9 × 10^-73^, which is the maximum probability attained at *βa *= 1209. The peak location *L*(*a*) is the extent from *x*_start _= 1204 to *x*_end _= 1216, which corresponds to the local minimum attained at *a *= 1212. The depth is *D*(*a*) = 0.711.

To simplify the presentation, we assume that *p*(*x*_1_) ≠ *p*(*x*_2_) if *x*_1 _≠ *x*_2_. Let Ψ be the set of *x*-values, where *p*(*x*) has local minima and maxima. We associate a possible peak with each element *a *∈ Ψ. If *a *is a local minimum, the peak is defined as illustrated in Figure 2. The *peak location *is the extent on the *x*-axis, *L*(*a*) = [*x*_start_(*a*), *x*_end_(*a*)], defined as the largest interval including *a *in which *p*(*x*) ≥ *p*(*a*). The *peak width *is the size of *L*(*a*), *p*_w_(*a*) = *x*_end_(*a*) - *x*_start_(*a*) + 1. The *peak volume *is the probability summed over the location, *p*_v_(*a*) = ∑_*x*∈*L*(*a*) _*p*(*a*). The peak's *bottom *(or *mode*) *βa *= arg max_*x*∈*L*(*a*) _*p*(*x*) is the *x*-value where *p *attains its maximum. (The term "bottom" originates from the corresponding energy landscape picture, but it is the position of the peak's top.) The *peak height *is *p*_h_(*a*) = *p*(*βa*). The peak's *depth *is $D(a)=\log_{10}\frac{p(\beta a)}{p(a)}$. We also associate a possible peak with each local maximum *a *∈ Ψ, namely the spike itself: *L*(*a*) = [*a*, *a*], *p*_w_(*a*) = 1, *βa *= *a*, *p*_v_(*a*) = *p*_h_(*a*) = *p*(*a*), and *D*(*a*) = 0.

While peaks may be high, it is a more defining characteristic that they are wide. A peak is produced by the fluctuations in *x*, rather than disturbed by them. For each local maximum, there are many possible peaks. Therefore, a peak can not be identified with its bottom. Instead, we use the elements in Ψ as unique identifiers of peaks. The location of a peak is *L*(*a*), not the bottom position *βa*, and the size of a peak is the peak volume, not the peak height. However, for the second type of peaks (the maxima), the peak location reduces to the bottom and the peak volume reduces to the peak height.

The set Ψ of possible peaks is hierarchically ordered. A binary tree is defined by the set inclusion order on the set of peak locations. For each pair *a, a' *∈ Ψ, either *L*(*a*) ⊆ *L*(*a'*), or *L*(*a*) ⊇ *L*(*a'*), or they are disjoint. The branching corresponds to each local minimum *a *dividing the peak into two subpeaks, see Figure 2, just as a barrier or a watershed or a saddle point divides two valleys or lakes in a landscape [36,38,39]. The global minimum is the *root *node *ρ *of the tree. The local maxima are the leaf nodes of the tree. Each *a *∈ Ψ has at most three edges, one towards the root and two away from the root. Each *a *≠ *ρ *has an edge towards the root that connects to the *successor σa*. Each successor has an increased depth: *D*(*σa*) ≥ *D*(*a*). And each local minimum *a *has two edges away from the root that connect to two *ancestors*. The highest peak of the two ancestors is the *father πa *and the other is the *mother μa*, i.e., they are distinguished by *p*_h_(*πa*) > *p*_h_(*μa*). A left-right distinction between the two is not used. The notation *σ*^*n *^*a *means the successor taken *n *≥ 0 times, where *σ*^0 ^*a *= *a*. Each *a *has a *set of successors *Σ(*a*) defined as the path from *a *to the root: *a*, *σa*, *σ*^2 ^*a*, ..., *ρ*. Each *a *also has a *set of ancestors *Δ(*a*) defined by *a' *∈ Δ(*a*) ⇔ *a *∈ Σ(*a'*). The set Δ(*a*) is the subtree that has *a *as its root node. A bottom is typically shared by several peaks. For example, a peak has the same bottom as its father, *βa *= *βπa*, but not the same as its mother, *βa *≠ *βμa*. Each *a *has a *paternal line *Π(*a*), defined as the set of all nodes that share *a*'s bottom. Π(*a*) is also the path including *a *connected by fathers that ends at *βa*. The beginning of the path, called the *full *node *φa*, is either a mother or the root. The paternal lines establish a one-to-one correspondence between the set of maxima (i.e. bottoms) and the set of mothers including the root.

Having established a hierarchy Ψ of possible peaks, the second step is to select among them. The selection applies two independent criteria, each controlled by an input parameter: the *maximum depth D*_max _and the *probability cutoff p*_c_. The first criterion is that *a *is a *1D peak *according to the following definition.

**Definition 1**. *Let D*_*max *_*be the maximum depth of peaks. Then a *∈ Ψ *is a *1D peak *if*

*(i) D*(*a*) <*D*_*max*_,

*(ii) D*(*σa*) ≥ *D*_*max*_*or a *= *ρ*.

The second criterion is that *p*_v_(*a*) ≥ *p*_c_. The first criterion is invoked by using the MAXDEEP subroutine [29], which returns the set *P *of all 1D peaks. The second criterion is subsequently invoked by calculating the peak volume of each *a *∈ *P *and comparing with the probability cutoff.

### Bubbles and helical regions

The stitch profile algorithm is separate from the statistical mechanical DNA melting model. The only interface to the underlying model is by calling the following probability functions:

(1) $$p_{\text{right}}(x)=P(\ldots \text{XX}\overset{x}{1}\underset{\text{unzipped}}{\underset{︸}{0\cdots 0}}-3^{\prime }),$$

(2) $$p_{\text{left}}(y)=P(5^{\prime }-\underset{\text{unzipped}}{\underset{︸}{0\cdots 0}}\overset{y}{1}\text{XX}\ldots ),$$

(3) $$p_{\text{bubble}}(x,y)=P(\ldots \text{XX}\overset{x}{1}\underset{\text{bubble}}{\underset{︸}{0\cdots 0}}\overset{y}{1}\text{XX}\ldots ),$$

(4) $$p_{\text{helix}}(x,y)=P(\ldots \text{XX0}\underset{\text{helix}}{\underset{︸}{\overset{x}{1}\cdots \overset{y}{1}}}\text{0XX}\ldots ),$$

(5) $$p_{\text{helix}}(x,N)=P(\ldots \text{XX0}\underset{\text{zipped}}{\underset{︸}{\overset{x}{1}\cdots 1}}-3^{\prime }),$$

(6) $$p_{\text{helix}}(1,y)=P(5^{\prime }-\underset{\text{zipped}}{\underset{︸}{1\cdots \overset{y}{1}}}0\text{XX}\ldots ).$$

In these equations, 1 is a bound basepair (helix), 0 is a melted basepair (coil), X is either 0 or 1, and the sequence positions *x *and/or *y *are indicated.

In addition to these, the stitch profile algorithm calls methods for adding these probabilites (peak volumes) and for computing upper bounds on such probability sums. This means that it is easy to change or replace the underlying model. In this article, the Poland-Scheraga model with Fixman-Freire loop entropies is used [30], but in principle, other DNA melting models could be used, or even models that include secondary structure [40].

This article discusses how to efficiently compute bubble stitches and helix stitches only. The 5' and 3' tail stitches are efficiently computed as in Algorithm 1 [29]. Each bubble stitch corresponds to a peak in the bubble probability function in Eq. (3). And each helix stitch corresponds to a peak in the helix probability function in Eq. (4). These two probability functions and their peaks are two dimensional, so the 1D peak finding method does not directly apply. However, the 1D peak analysis can be performed for each of the other four probability functions [Eqs. (1), (2), (5), and (6)]. Using Eq. (1), a binary tree Ψ_x _and a set of 1D peaks *P*_x _is computed, and using Eq. (2), a binary tree Ψ_y _and a set of 1D peaks *P*_y _is computed. The probability cutoff is not invoked here. These two tree structures with their 1D peaks are then further processed, as described in the following two sections, to obtain the bubble stitches. Likewise, using Eq. (5), a binary tree Ψ_x _and a set of 1D peaks *P*_x _is computed, and using Eq. (6), a binary tree Ψ_y _and a set of 1D peaks *P*_y _is computed. These are used similarly to obtain the helix stitches. This division of labor also indicates an obvious parallelization of the algorithm using two or four processors. Parallelism was not implemented in this study, however.

### 2D peaks

The goal of this section is to define 2D peaks and to prove the key result that some 2D peaks are simply the Cartesian product of two 1D peaks. But not all 2D peaks have this property, making it a nontrivial result. This is expressed in Theorem 2.

Theorem 2 also indicates a convenient way of computing all 2D peaks, on which Algorithm 2 is directly based. Theorem 2 shows that Algorithm 2's computation of stitch profiles is exact, that is, complying strictly with the mathematical definition of 2D peaks. The proof is therefore important for the validation of Algorithm 2. While Theorem 2 is the primary goal, we also prove Theorem 1 which similarly provides validation of Algorithm 1. But more importantly, a comparison of the two theorems gives more insight in both algorithms.

A *frame *is a pair (*a*, *b*) ∈ Ψ_x _× Ψ_y_. A frame also refers to the corresponding box *L*(*a*) × *L*(*b*) in the *xy*-plane. A frame (*a*, *b*) is *contained inside *another frame (*a'*, *b'*), if *L*(*a*) × *L*(*b*) ⊂ *L*(*a'*) *× L*(*b'*), that is, if *a' *∈ Σ(*a*) and *b' *∈ Σ(*b*). The *root frame *is (*ρ*_x_, *ρ*_y_). A frame (*a*, *b*) is *nonroot *if (*a*, *b*) ≠ (*ρ*_x_, *ρ*_y_). A frame (*a*, *b*) is a *bottom frame *if (*a*, *b*) = (*βa*, *βb*) and it is *nonbottom *if (*a*, *b*) ≠ (*βa*, *βb*). The *depth *of a frame (*a*, *b*) is *D*(*a*, *b*) = max{*D*(*a*), *D*(*b*)}. From this definition, we immediately get

(7) *D*(*a*, *b*) <*D*_max _⇔ *D*(*a*) <*D*_max _and *D*(*b*) <*D*_max_.

To simplify the presentation, we assume that for all frames: *D*(*a*) ≠ *D*(*b*).

**Definition 2**. *The *successor *of a nonroot frame *(*a*, *b*) *is*

(8) $$\sigma (a,b)=\{\begin{array}{l} (\sigma a,b)\;if\;D(\sigma b)>D(\sigma a)\;or\;b=\rho_{y}\\ (a,\sigma b)\;if\;D(\sigma a)>D(\sigma b)\;or\;a=\rho_{x} \end{array}$$

*A successor of the root frame does not exist*.

Having defined the depth and the successor, what is the depth of a successor?

**Proposition 1**. *For every nonroot *(*a*, *b*), *D*(*σ*(*a*, *b*)) ≥ *D*(*a*, *b*).

*Proof*. For *σ*(*a*, *b*) = (*σa*, *b*), max{*D*(*σa*), *D*(*b*)} ≥ max{*D*(*a*), *D*(*b*)} because *D*(*σa*) ≥ *D*(*a*). Likewise for *σ*(*a*, *b*) = (*a*, *σb*).   □

**Definition 3**. *A frame *(*a*, *b*) *is σ*-above *if*

*(i) D*(*σa*) > *D*(*b*) *or a *= *ρ*_*x*_,

*(ii) D*(*σb*) > *D*(*a*) *or b *= *ρ*_*y*_.

The term "*σ*-above" is a mnemonic for the two inequalities in the definition. The set of all frames that are *σ*-above is called *the frame tree*. While Prop. 1 only sets a lower bound on the depth of a successor, we can write the actual value for *σ*-above frames:

**Proposition 2**. *If *(*a*, *b*) *is nonroot and σ-above, then*

(9) $$D(\sigma (a,b))=\{\begin{array}{l} D(\sigma a)\;if\;\sigma (a,b)=(\sigma a,b)\\ D(\sigma b)\;if\;\sigma (a,b)=(a,\sigma b). \end{array}$$

*Furthermore, D*(*σ*(*a*, *b*)) = *min*{*D*(*σa*), *D*(*σb*)} *if both a *≠ *ρ*_*x *_*and b *≠ *ρ*_*y*_.

*Proof*. If *σ*(*a*, *b*) = (*σa*, *b*), then *a *≠ *ρ*_x _and max{*D*(*σa*), *D*(*b*)} = *D*(*σa*) by Def. 3. If, furthermore, *b *≠ *ρ*_y_, then *D*(*σ*(*a*, *b*)) = *D*(*σa*) <*D*(*σb*) by Def. 2. Likewise if *σ*(*a*, *b*) = (*a*, *σb*).   □

By repeatedly taking the successor, we eventually end up at the root frame in, say, *R *steps. Σ(*a*, *b*) is the *sequence of successors *of (*a*, *b*), i.e., the sequence ${\left\{\sigma^{n}(a,b)\right\}}_{0}^{R}$ that begins at (*a*, *b*) and ends at the root frame. Alternatively, Σ(*a*, *b*) is defined as the *set of successors*, i.e., the set of such sequence elements. What if we want to exclude (*a*, *b*) from Σ(*a*, *b*)? That can be written as Σ(*σ*(*a*, *b*)).

If (*a*, *b*) is not *σ*-above, then its sequence of successors takes the shortest path to a *σ*-above frame, or put another way:

**Proposition 3**. *If a' *∈ Σ(*a*), *b' *∈ Σ(*b*) *and *(*a'*, *b'*) *is σ-above, then *(*a'*, *b'*) ∈ Σ(*a*, *b*).

*Proof*. All elements in both Σ(*a*) and Σ(*b*) are visited by the sequence Σ(*a*, *b*) on its climb to the root frame. Assume (*a'*, *b'*) ∉ Σ(*a*, *b*). Then either *a' *is passed before *b' *is reached, or viceversa, and we can assume that *a' *comes first. In other words, *a' *≠ *ρ*_x _and there is a *b" *≠ *b' *such that *b' *∈ Σ(*b"*) and *σ*(*a'*, *b"*) = (*σa'*, *b"*). Then *D*(*b'*) ≥ *D*(*σb"*). By Def. 2, we see that *D*(*σb"*) > *D*(*σa'*). (*a'*, *b'*) is *σ*-above, so by Def. 3, we see that *D*(*σa'*) > *D*(*b'*). We arrive at the contradiction *D*(*b'*) > *D*(*b'*).   □

Each frame is the successor of at most four frames. If (*a*, *b*) = *σ*(*a'*, *b'*) then (*a'*, *b'*) is either (*πa*, *b*), (*a*, *πb*), (*μa*, *b*), or (*a*, *μb*). Two of these are defined as *ancestors*:

**Definition 4**. *The *father *of a nonbottom frame *(*a*, *b*) *is*

(10) $$\pi (a,b)=\{\begin{array}{l} \left(\pi a,b)\;if\;D(a)>D(b\right)\\ \left(a,\pi b)\;if\;D(a)<D(b\right) \end{array}$$

*The *mother *of a nonbottom frame *(*a*, *b*) *is*

(11) $$\mu (a,b)=\{\begin{array}{l} \left(\mu a,b)\;if\;D(a)>D(b\right)\\ (a,\mu b)\;if\;D(a)<D(b). \end{array}$$

*Fathers and mothers of bottom frames do not exist*.

Each father or mother can have its own father and mother, and so on. The *set of ancestors *Δ(*a*, *b*) is the binary subtree defined recursively by: (1) (*a*, *b*) ∈ Δ (*a*, *b*). (2) If nonbottom (*a'*, *b'*) ∈ Δ(*a*, *b*) then *π*(*a'*, *b'*) ∈ Δ(*a*, *b*) and *μ*(*a'*, *b'*) ∈ Δ(*a*, *b*).

The next proposition shows that being *σ*-above is propagated by *σ*, *π*, and *μ*:

**Proposition 4**. *Let *(*a*, *b*) *be σ-above*.

*(i) If *(*a'*, *b'*) ∈ Σ(*a*, *b*) *then *(*a'*, *b'*) *is σ-above*.

*(ii) If *(*a'*, *b'*) ∈ Δ(*a*, *b*) *then *(*a'*, *b'*) *is σ-above*.

*Proof*. (i): First, we show that *σ*(*a*, *b*) is *σ*-above: If *σ*(*a*, *b*) = (*σa*, *b*), then Def. 2 implies the second condition: *D*(*σb*) > *D*(*σa*) or *b *= *ρ*_y_. And (*a*, *b*) is *σ*-above which by Def. 3 implies the first condition: *D*(*σ*^2^*a*) > *D*(*σa*) > *D*(*b*) or *σa *= *ρ*_x_. Similarly, *σ*(*a*, *b*) = (*a*, *σb*) is shown to be *σ*-above. The proof is completed by induction.

(ii): First, we show that *π*(*a*, *b*) is *σ*-above: If *π*(*a*, *b*) = (*πa*, *b*), then Eq. (10) implies the first condition: *D*(*σπa*) = *D*(*a*) > *D*(*b*) or *πa *= *ρ*_x_. And (*a*, *b*) is *σ*-above which by Def. 3 implies the second condition: *D*(*σb*) > *D*(*a*) > *D*(*πa*) or *b *= *ρ*_y_. Similarly, *π*(*a*, *b*) = (*a*, *πb*) and *μ*(*a*, *b*) are shown to be *σ*-above. The proof is completed by induction.   □

Successors are the inverse of fathers and/or mothers for *σ*-above frames only:

**Proposition 5**. *If *(*a*, *b*) *is nonbottom and nonroot, the following statements are equivalent:*

*(i) *(*a*, *b*) *is σ-above*

*(ii) σπ*(*a*, *b*) = (*a*, *b*)

*(iii) σμ*(*a*, *b*) = (*a*, *b*)

*(iv) πσ*(*a*, *b*) = (*a*, *b*) *or μσ*(*a*, *b*) = (*a*, *b*)

*Proof*. (*i*) ⇔ (*ii*): If *π*(*a*, *b*) = (*πa*, *b*), then Eq. (10) implies the first condition that (*a*, *b*) is *σ*-above: *D*(*σa*) > *D*(*a*) > *D*(*b*) or *a *= *ρ*_x_. Then (*a*, *b*) is *σ*-above $\overset{\text{Def}.3}{\Leftrightarrow }$*D(σb)* > *D*(*a*) = *D*(*σπa*) or *b *= *ρy* = $\overset{\text{Def}.2}{\Leftrightarrow }$*σ(πa,b)* = (*σπa*, *b*) ⇔ *σπ*(*a*, *b*) = (*a*, *b*). If *π*(*a*, *b*) = (*a*, *πb*), the equivalence is shown similarly.

(*i*) ⇔ (*iii*): Replace *π *by *μ *in the above.

(*i*) ⇔ (*iv*): If *σ*(*a*, *b*) = (*σa*, *b*), then Def. 2 implies the second condition that (*a*, *b*) is *σ*-above:* D*(*σb*) > *D*(*σa*) > *D*(*a*) or *b *= ρ_y_. Then (*a*, *b*) is *σ*-above $\overset{\text{Def}.3}{\Leftrightarrow }$*D*(*σa*) > *D*(*b*) $\overset{\text{Def}.4}{\Leftrightarrow }$*π(σa*, *b*) = (*πσa*, *b*) or *μ*(*σa*, *b*) = (*μσa*, *b*) ⇔ *π(σa, b) = (πσa, b) or  µ(σa, b) = (µσa, b) *⇔* πσ(a, b) = (a, b) or µσ(a, b) = (a, b).* If *σ*(*a*, *b*) = (*a*, *σb*), the equivalence is shown similarly.   □

Accordingly, there is an "inverse" relationship between the sets of successors and ancestors:

**Proposition 6**. (*a'*, *b'*) *is σ-above and *(*a*, *b*) ∈ Σ(*a'*, *b'*) iff (*a*, *b*) *is σ-above and *(*a'*, *b'*) ∈ Δ(*a*, *b*).

*Proof*. (*a*, *b*) ∈ Σ(*a'*, *b'*) implies a path of successors from (*a'*, *b'*) to (*a*, *b*). Prop. 4 shows that all elements in the path are *σ*-above. Prop. 5(iv) applied to each step in the path gives an opposite path of ancestors.

Conversely, (*a'*, *b'*) ∈ Σ(*a*, *b*) implies a path of ancestors from (*a*, *b*) to (*a'*, *b'*). Prop. 4 shows that all elements in the path are *σ*-above. Prop. 5(ii) and (iii) applied to each step in the path gives an opposite path of successors.   □

It follows from Prop. 6 that the frame tree is equal to the binary tree Δ(*ρ*_x_, *ρ*_y_), because (*ρ*_x_, *ρ*_y_) ∈ Σ(*a'*, *b'*) for any (*a'*, *b'*). It has the same pedigree properties as Ψ, such as paternal lines and *βπ*(*a*, *b*) = *β*(*a*, *b*). So far, we have covered ground that was already implicit in [29], but augmented here with proofs. The next concept is new, however, namely the Cartesian products of 1D peaks.

**Definition 5**. (*a*, *b*) *is a *grid frame *if a and b are 1D peaks*.

**Figure 3:**
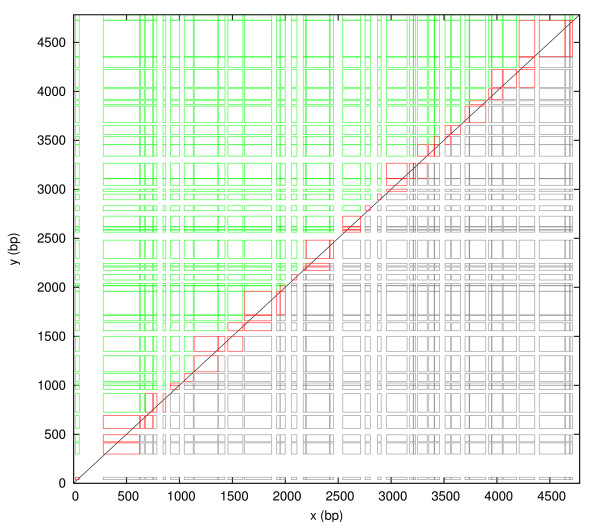
**The set *G *= *P*_**x **_× *P*_**y **_of all grid frames plotted in the *xy*-plane**. The grid frames are colored to distinguish those that are above the diagonal (green), crossing the diagonal (red), and below the diagonal (grey), thus illustrating the subsets *G*_a_, *G*_c _and *G*_b_, respectively. Frames with side lengths below 20 bp are not shown to unclutter the figure.

The set of all grid frames is *G *= *P*_x _*× P*_y_. As Figure 3 shows, *G *has a grid-like ordering in the *xy*-plane. All 1D peaks *a *∈ *P*_x _have disjoint peak locations *L*(*a*) = [*x*_start_(*a*), *x*_end_(*a*)]. They can be indexed by *i *= 1, 2, 3, ... according to their ordering from 5' to 3' on the sequence, such that *x*_end_(*a*_*i*_) <*x*_start_(*a*_*i*+1_). Likewise, the 1D peaks *b *∈ *P*_y _can be indexed by *j*. Then the grid frames form a matrix *G *with elements [*G*]_*ij *_= (*a*_*i*_, *b*_*j*_). We use the symbol *G *for both the set and the matrix.

**Proposition 7**. *Every grid frame *(*a*, *b*) *is σ-above*.

*Proof*. If *a *≠ *ρ*_x_, then *D*(*σa*) ≥ *D*_max _because *a *is a 1D peak and *D*_max _> *D*(*b*) because *b *is a 1D peak (see Def. 1), thus showing Def. 3(i). Similarly, we show Def. 3(ii).   □

The following two lemmas show that grid frames inherit some properties from 1D peaks.

**Lemma 1**. (*a*, *b*) *is a grid frame *iff

*(i) *(*a*, *b*) *is σ-above*,

*(ii) D*(*a*, *b*) <*D*_*max*_,

*(iii) D*(*σ*(*a*, *b*)) ≥ *D*_*max *_*or *(*a*, *b*) *is the root frame*.

*Proof*. If (*a*, *b*) is a grid frame, then it is *σ*-above by Prop. 7 and Eq. (7) implies *D*(*a*, *b*) <*D*_max_. For nonroot (*a*, *b*), *D*(*σ*(*a*, *b*)) equals either *D*(*σa*) or *D*(*σb*) (Prop. 2), which is ≥ *D*_max _because *a *and *b *are 1D peaks.

Conversely, Eq. (7) implies *D*(*a*) <*D*_max_. For *a *= *ρ*_x_, *a *is then a 1D peak. For *a *≠ *ρ*_x_, Prop. 2 gives *D*(*σa*) ≥ *D*(*σ*(*a*, *b*)) ≥ *D*_max_, so *a *is a 1D peak. Similarly, *b *is shown to be a 1D peak.   □

**Lemma 2**. *Let D*_*max*_*be the maximum depth of peaks*.

*(i) For each a with D*(*a*) <*D*_*max*_, *there is exactly one 1D peak a' *∈ Σ(*a*).

*(ii) For each *(*a*, *b*) *with D*(*a*, *b*) <*D*_*max*_, *there is exactly one grid frame *(*a'*, *b'*) ∈ Σ(*a*, *b*).

*Proof*. (i): The depth increases monotonically in the sequence Σ(*a*) of successors (∀*n *: *D*(*σ*^*n*^*a*) ≤ *D*(*σ*^*n*+1^*a*)). For *D*(*ρ*_x_) ≥ *D*_max_, there is therefore a unique element *a' *≠ *ρ*_x _with *D*(*a'*) <*D*_max _and *D*(*σa'*) ≥ *D*_max_. For *D*(*ρ*_x_) <*D*_max_, *a' *= *ρ*_x _is a 1D peak and no other element in Σ(*a*) can fulfill Def. 1(ii).

(ii): Eq. (7) gives *D*(*a*) <*D*_max _and *D*(*b*) <*D*_max_. By applying (i) to *a *and *b*, we obtain a unique grid frame (*a'*, *b'*) where *a' *∈ Σ(*a*) and *b' *∈ Σ(*a*). (*a'*, *b'*) is *σ*-above by Prop. 7, so (*a'*, *b'*) ∈ Σ(*a*, *b*) by Prop. 3.   □

How do we define 2D peaks? A straightforward way would be to generalize 1D peaks by simply rewriting Def. 1 in the frame tree context. The result would be the grid frames, as we see by Lemma 1. However, there is more to the picture than the frame tree, due to a further constraint to be discussed next, which requires a more elaborate definition of 2D peaks.

In genomic annotations, a region is specified by coordinates *x..y*, where by convention *x *<*y*, i.e., *x *is the 5' end and *y *is the 3' end. We adopt the same constraint for our notation (*x*, *y*) of the instantaneous location of a bubble or helix. In the *xy*-plane, helices are only defined for (*x*, *y*) above the diagonal line *y *= *x*. Bubbles have at least one melted basepair in between *x *and *y*, so they are only defined for (*x*, *y*) above the diagonal line *y *= *x *+ 1. Accordingly, we require that frames are above the diagonal line, as defined in the following.

**Definition 6**. *A frame *(*a*, *b*) *is *above the diagonal *if*

(12a) *x*_*end*_(*a*) + 1 <*y*_*start*_(*b*) *for bubbles*,

(12b) *x*_*end*_(*a*) <*y*_*start*_(*b*) *for helices*.

*A frame *(*a*, *b*) *is *below the diagonal *if*

(13a) *x*_*start*_(*a*) + 1 ≥ *y*_*end*_(*b*) *for bubbles*,

(13b) *x*_*start*_(*a*) ≥ *y*_*end*_(*b*) *for helices*.

*A frame *(*a*, *b*) *is *crossing the diagonal *if it is neither above the diagonal nor below the diagonal*.

Note: A frame that is crossing the diagonal contains at least one point (*x*, *y*) above the diagonal line, while a frame that is below the diagonal contains no points above the diagonal line, but its upper left corner may be on the diagonal line. Figure 3 illustrates frames that are above, crossing and below the diagonal.

The requirement that a frame is above the diagonal puts a constraint on its size. This is embodied in the next concept.

**Definition 7**. *The root frame is a *fractal frame *if it is above the diagonal. A nonroot frame *(*a*, *b*) *is a *fractal frame *if*

*(i) *(*a*, *b*) *is above the diagonal*,

*(ii) σ*(*a*, *b*) *is crossing the diagonal*,

*(iii) *(*a*, *b*) *is σ-above*.

**Figure 4:**
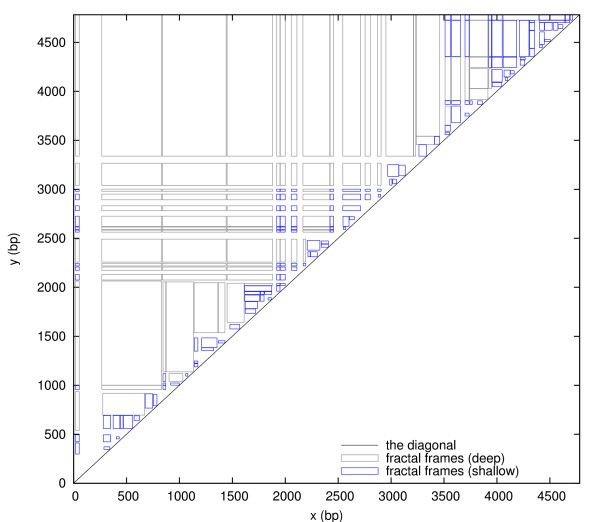
**The set *F *of all fractal frames plotted in the *xy*-plane**. The fractal frames (*a*, *b*) ∈ *F *are colored to distinguish those with depths *D*(*a*, *b*) ≥ *D*_max _(grey) and *D*(*a*, *b*) <*D*_max _(blue), thus illustrating the subsets *F*_d _and *F*_s_, respectively. Frames with side lengths below 20 bp are not shown to unclutter the figure.

The set of all fractal frames is denoted *F*. As Figure 4 shows, fractal frames tend to be smaller the closer they are to the diagonal, thus resembling a fractal. For a typical fractal frame, the fluctuations in *x *and *y *are comparable in size to the length *y *- *x *of the bubble or helix itself. Indeed, the two peak locations *L*(*a*) and *L*(*b*) are as wide as possible, while not overlapping each other (because the successor is crossing the diagonal). In contrast, the fluctuations for grid frames are relatively small on average and independent of the bubble or helix length.

**Lemma 3**. *For each σ-above and above the diagonal *(*a*, *b*), *there is exactly one fractal frame *(*a'*, *b'*) ∈ Σ(*a*, *b*).

*Proof*. Let (*a'*, *b'*) = *σ*^*n*^(*a*, *b*), where *n *is the largest number for which *σ*^*n*^(*a*, *b*) is above the diagonal. (*a'*, *b'*) is *σ*-above by Prop. 4. For all *m *> *n*, frames *σ*^*m*^(*a*, *b*) (if they exist) are not above the diagonal, nor below the diagonal because they contain (*a*, *b*), hence they are crossing the diagonal. Therefore (*a'*, *b'*) is a fractal frame. For all *m *<*n*, frames *σ*^*m*^(*a*, *b*) (if they exist) are above the diagonal, because they are contained in (*a'*, *b'*). Therefore (*a'*, *b'*) is the only fractal frame in Σ(*a*, *b*).   □

Lemma 3 is similar to Lemma 2. By Prop. 6, we can express both lemmas in terms of ancestors Δ instead of successors Σ. The lemmas then say that certain kinds of frames are organized as forests. A *forest *is a set of disjoint trees. The sets *F *and *G *generate two forests: ∪_(*a*, *b*) ∈ *G *_Δ(*a*, *b*) consists of the subtrees having grid frames as root nodes. ∪_(*a*,*b*) ∈ *F *_Δ(*a*, *b*) consists of the subtrees having fractal frames as root nodes. By these forests, we generate from *G *the set of all *σ*-above frames with *D*(*a*, *b*) <*D*_max_, and we generate from *F *the set of all *σ*-above frames above the diagonal.

All the necessary concepts are now in place for the definition of 2D peaks. We will not repeat the "derivation" of 2D peaks given in [29], but just recall that 2D peaks are defined with a purpose: They must capture the extent of the actual peaks in the probability functions *p*_bubble_(*x*, *y*) and *p*_helix_(*x*, *y*). And they must have an interpretation in terms of fluctuations on a given timescale. The following definition is equivalent to the formulation in [29].

**Definition 8**. *Let D*_*max *_*be the maximum depth of peaks. A frame *(*a*, *b*) *is a *2D peak *if*

*(i) *(*a*, *b*) *is above the diagonal*,

*(ii) *(*a*, *b*) *is σ-above*,

*(iii) D*(*a*, *b*) <*D*_*max*_,

*(iv) D*(*σ*(*a*, *b*)) ≥ *D*_*max *_*or *(*a*, *b*) *is a fractal frame*.

**Figure 5:**
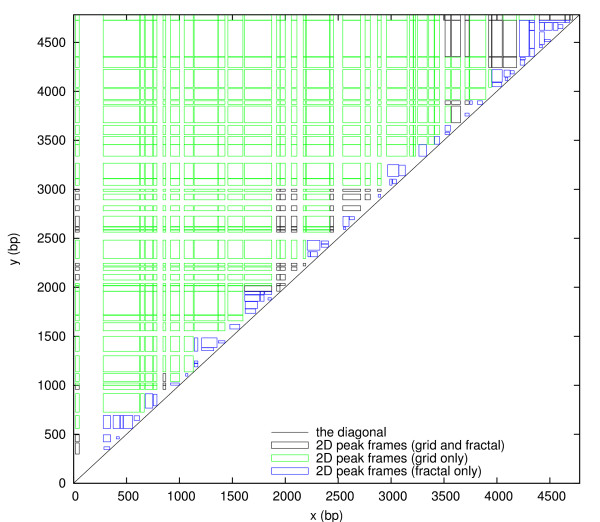
**The set *P *of all 2D peaks plotted in the *xy*-plane**. The 2D peak frames are colored to distinguish those that are fractal frames (blue), fractal frames and grid frames (black), or grid frames (green), thus illustrating the subsets *P*_F_, *P*_FG _and *P*_G_, respectively. Frames with side lengths below 20 bp are not shown to unclutter the figure.

Note: the *or *in the definition is not an *exclusive or*. A 2D peak (*a*, *b*) can both be a fractal frame *and *have *D*(*σ*(*a*, *b*)) ≥ *D*_max_. The set of all 2D peaks is denoted *P *and is illustrated in Figure 5.

Comparing Def. 8 and Lemma 1, we see that the difference between 2D peaks and grid frames is due to the diagonal constraint: First, the requirement that 2D peaks are above the diagonal, and second, the possible exemption from the second inequality, which for grid frames is being the root frame, while for 2D peaks it is being a fractal frame. Unlike grid frames, 2D peaks can capture events close to the diagonal by adapting their size.

Computing the 2D peaks is at the core of the stitch profile methodology. The following two theorems provide characterizations of 2D peaks that may be translated into computer programs.

**Theorem 1**. *We divide 2D peaks into two types, being fractal frames or not, that can be distinctly characterized as follows*.

*(i) *(*a*, *b*) *is a 2D peak and a fractal frame *iff (*a*, *b*) *is a fractal frame and D*(*a*, *b*) <*D*_*max*_.

*(ii) *(*a*, *b*) *is a 2D peak and not a fractal frame *iff (*a*, *b*) *is a grid frame and there is a fractal frame *(*a'*, *b'*) *with D*(*a'*, *b'*) ≥ *D*_*max*_, *such that *(*a'*, *b'*) ∈ Σ(*a*, *b*).

*Proof*. (i): Immediate by Defs. 7 and 8.

(ii): If a 2D peak (*a*, *b*) is not a fractal frame, then *D*(*σ*(*a*, *b*)) ≥ *D*_max _by Def. 8, so (*a*, *b*) is a grid frame by Lemma 1. Applying Lemma 3, there is a fractal frame (*a'*, *b'*) ∈ Σ(*a*, *b*). (*a*, *b*) ≠ (*a'*, *b'*) because one is a fractal frame, the other is not, so (*a'*, *b'*) ∈ Σ(*σ*(*a*, *b*)), which by Prop. 1 implies *D*(*a'*, *b'*) ≥ *D*_max_.

Conversely, (*a*, *b*) is above the diagonal because it is contained in a fractal frame. (*a*, *b*) ≠ (*a'*, *b'*) because *D*(*a*, *b*) <*D*_max _and *D*(*a'*, *b'*) ≥ *D*_max_, implying that (*a*, *b*) is not a fractal frame (uniqueness by Lemma 3) and not the root frame. The other requirements for a 2D peak are established by Lemma 1.   □

Theorem 1 characterizes all 2D peaks by their relationship to fractal frames. This is applied in Algorithm 1, that derives all 2D peaks from fractal frames. However, the next theorem shows that some 2D peaks can be characterized without referring to fractal frames.

**Theorem 2**. *A nonroot 2D peak has a successor, the depth of which is either greater or less than D*_*max*_. *We thus divide 2D peaks into two types, that can be distinctly characterized as follows. Let *(*a*, *b*) *be nonroot. Then*

*(i) *(*a*, *b*) *is a 2D peak and D*(*σ*(*a*, *b*)) ≥ *D*_*max *_iff (*a*, *b*) *is a grid frame that is above the diagonal*.

*(ii) *(*a*, *b*) *is a 2D peak and D*(*σ*(*a*, *b*)) <*D*_*max *_iff (*a*, *b*) *is a fractal frame and there is a grid frame *(*a'*, *b'*) *that is crossing the diagonal, such that *(*a'*, *b'*) ∈ Σ(*a*, *b*).

*Proof*. (i): Immediate by Def. 8 and Lemma 1.

(ii): If a 2D peak (*a*, *b*) has *D*(*σ*(*a*, *b*)) <*D*_max_, then (*a*, *b*) is a fractal frame by Def. 8. Applying Lemma 2 to *σ*(*a*, *b*), there is a grid frame (*a'*, *b'*) ∈ Σ(*σ*(*a*, *b*)) ⊂ Σ(*a*, *b*). Frame (*a'*, *b'*) is crossing the diagonal because it contains *σ*(*a*, *b*), which is crossing the diagonal because (*a*, *b*) is a fractal frame.

Conversely, (*a*, *b*) ≠ (*a'*, *b'*) because (*a*, *b*) is above the diagonal (a fractal frame) and (*a'*, *b'*) is crossing the diagonal, and hence (*a'*, *b'*) ∈ Σ(*σ*(*a*, *b*)). Since (*a'*, *b'*) is a grid frame, Lemma 1 gives *D*(*a'*, *b'*) <*D*_max_, which by Prop. 1 implies *D*(*a*, *b*) ≤ *D*(*σ*(*a*, *b*)) <*D*_max_, and we conclude that (*a*, *b*) is a 2D peak.   □

Note: Theorem 2 does not consider the root frame. However, if the root frame is a 2D peak, then it is of the first type: a grid frame that is above the diagonal.

It follows from Theorems 1 and 2 that a 2D peak is either a grid frame, a fractal frame, or both. The set of 2D peaks *P *can therefore be divided into three disjoint sets defined as follows. *P*_F _are the 2D peaks that are fractal frames only, not grid frames. *P*_FG _are the 2D peaks that are both fractal frames and grid frames. *P*_G _are the 2D peaks that are grid frames only, not fractal frames. Let *G*_a_, *G*_b _and *G*_c _be the sets of grid frames that are above, below and crossing the diagonal, respectively. Let *F*_d _and *F*_s _be the sets of fractal frames that are deep (*D*(*a*, *b*) ≥ *D*_max_) and shallow (*D*(*a*, *b*) <*D*_max_), respectively. In Figs. 3, 4, 5, all these subsets are illustrated with different colors. The following corollary summarizes the relationships between grid frames, fractal frames and 2D peaks:

**Corollary 1**. *The set of 2D peaks is P *= *F*_*s *_∪ *G*_*a*_. *The intersection between the grid and the fractal is P*_*FG *_= *F*_*s *_∩ *G*_*a *_= *F *∩ *G. Furthermore, the 2D peaks can be obtained by the following two expressions, in which all set unions are between disjoint sets:*

(14a) $$P=F_{s}\underset{(a^{\prime },b^{\prime })\in F_{d}}{\cup }G\cap \Delta (a^{\prime },b^{\prime }),$$

(14b) $$=G_{a}\underset{(a^{\prime },b^{\prime })\in G_{c}}{\cup }F\cap \Delta (a^{\prime },b^{\prime }).$$

*Proof. P *= *P*_F _∪ *P*_FG _∪ *P*_G_. Theorem 1 states that *P*_F _∪ *P*_FG _= *F*_s _and that   

$$P_{G}=\underset{(a^{\prime },b^{\prime })\in F_{d}}{\cup }G\cap \Delta (a^{\prime },b^{\prime }).$$

Here, Δ(*a'*, *b'*) is brought into play by Prop. 6. Theorem 2 states that *P*_FG _∪ *P*_G _= *G*_a _(the root frame would go here) and that

$$P_{F}=\underset{(a^{\prime },b^{\prime })\in G_{c}}{\cup }F\cap \Delta (a^{\prime },b^{\prime }).\;\;□$$

Eqs. (14a) and (14b) outline how the set of 2D peaks is built up computationally by Algorithm 1 and 2, respectively. Writing the expressions side by side shows the parallels: Algorithm 1 takes some fractal frames and then it adds some grid frames that are contained inside fractal frames. Algorithm 2 takes some grid frames and then it adds some fractal frames that are contained inside grid frames. In both cases, the additional part is the more complicated part, as it requires searching some forests. The two Algorithms are algorithmically equivalent in terms of output, but the transformation in Eq. (14) from *F *-based to *G*-based facilitates a reduction in execution time, as described in the next section.

### The fast and exact algorithm

Algorithm 2 owes its speed to two important ingredients: One is the grid frame matrix *G *associated to the parameter *D*_max_. The other is an upper bound associated to the parameter *p*_*c*_.

To compute all bubble stitches of the stitch profile, the algorithm must find those 2D peaks (*a*, *b*) in the bubble context that have a peak volume

(15) $$p_{\text{v}}(a,b)=\sum_{x\in L(a)}\sum_{y\in L(b)}p_{\text{bubble}}(x,y)$$

that is greater or equal to the probability cutoff *p*_*c*_. According to Eq. (14b), one can write an algorithm for obtaining all 2D peaks using two nested loops that goes through all matrix elements (*a*_*i*_, *b*_*j*_) of the grid frame matrix *G*: If (*a*_*i*_, *b*_*j*_) is above the diagonal, it is a 2D peak. If (*a*_*i*_, *b*_*j*_) is crossing the diagonal, a subroutine computes the set *F *∩ Δ(*a*_*i*_, *b*_*j*_). If (*a*_*i*_, *b*_*j*_) is below the diagonal, it is skipped. By piping the resulting frames through a probability cutoff filter, we obtain the bubble stitches.

The matrix *G *is not stored in memory, only the two arrays *P*_x _and *P*_y _that provide each *a*_*i *_and *b*_*j*_. Matrix elements (*a*_*i*_, *b*_*j*_) being above, crossing or below the diagonal refers to the diagonal line in the *xy*-plane, never the diagonal of the matrix. For each row and column of the matrix there may be zero, one, or more matrix elements that are crossing the diagonal, as can be seen in Figure 3.

More specifically, let *G *be of order *m × n *and let the outer loop be over *j *= *n *to 1 and the inner loop over *i *= *m *to 1. The iteration thus begins at the upper right corner of Figure 3 and steps along the *y*-axis in the outer loop and the *x*-axis in the inner loop. However, we do not have to start at *i *= *m *for each *j*. If (*a*_*i*_, *b*_*j*_) is below the diagonal, then (*a*_*i*_, *b*_*k*_) is below the diagonal for all *k < j*. Therefore, we can jump directly to the *i *that corresponds to the first grid frame that was not below the diagonal at the previous *j*. In this way, most of the grid frames that are below the diagonal are ignored by the algorithm. While this is a trivial programming trick, we shall now see a less trivial trick, that ignores most of the grid frames that are above the diagonal.

Recall [30] that the bubble probability is

(16) $$p_{\text{bubble}}(a,b)=\frac{Z_{\text{X}10}(x)\Omega (y-x)Z_{01\text{X}}(y)}{Z}.$$

The loop entropy factor Ω(*y *- *x*) is a monotonically decreasing function. Its largest value in a frame (*a*, *b*) is therefore in the lower right corner, i.e. Ω_max _= Ω(*y*_start_(*b*) - *x*_end_(*a*)). Then

$$p_{\text{v}}(a,b)\leq \frac{\Omega_{\max}}{Z}\left(\sum_{x\in L(a)}Z_{\text{X}10}(x)\right)\left(\sum_{y\in L(b)}Z_{01\text{X}}(y)\right)$$

and the bubble peak volume has an upper bound that factorizes. Using the 1D peak volumes

(17a) $$p_{\text{v}}(a)=\sum_{x\in L(a)}Z_{\text{X}10}(x)/Z,$$

(17b) $$p_{\text{v}}(b)=\sum_{y\in L(b)}Z_{01\text{X}}(y)/Z,$$

we can write the upper bound as

(18) $${\tilde{p}}_{\text{v}}(a,b)=\Omega (y_{\text{start}}(b)-x_{\text{end}}(a))Zp_{\text{v}}(a)p_{\text{v}}(b).$$

If a grid frame (*a*_*i*_, *b*_*j*_) has an upper bound below the cutoff, ${\tilde{p}}_{\text{v}}$(*a*_*i*_, *b*_*j*_) <*p*_*c*_, then also ${\tilde{p}}_{\text{v}}$(*a*_*k*_, *b*_*j*_) <*p*_*c*_for all *k < i *for which *p*_v_(*a*_*k*_) ≤ *p*_v_(*a*_*i*_), because the loop entropy factor is decreasing. In that case, their peak volumes are also below the cutoff, of course, and the algorithm can reject all these frames. We implement this observation by calculating in advance the *next bigger goat *nbg(*i*) defined by

(i) *p*_v _(*a*_*k*_) ≤ *p*_v _(*a*_*i*_) for nbg(*i*) <*k < i*

(ii) *p*_v_(*a*_nbg(*i*)_) > *p*_v_(*a*_*i*_)

The nbg(*i*) is calculated as follows: A loop over *i *= 1 to *m *compares each *p*_v_(*a*_*i*_) successively to *p*_v_(*a*_*i*-1_), *p*_v_(*a*_nbg(*i*-1)_), *p*_v _(*a*_nbg(nbg(*i*-1))_), ... until a bigger one is found or the list ends.

For grid frames (*a*_*i*_, *b*_*j*_) that are above the diagonal, the algorithm first checks if ${\tilde{p}}_{\text{v}}$ (*a*_*i*_, *b*_*j*_) <*p*_*c*_, in which case it jumps directly to (*a*_nbg(*i*)_, *b*_*j*_). The nbg(*i*) may be undefined, if there are no bigger *p*_v_(*a*_*k*_), in which case the inner loop is done and the outer loop proceeds to the next *j*. On the other hand, if ${\tilde{p}}_{\text{v}}$ (*a*_*i*_, *b*_*j*_) ≥ *p*_*c*_, then the peak volume has to be calculated and checked. Although grid frames may be skipped without having calculated neither their peak volumes nor their upper bounds, the criterion for rejection is exact. There are no false negatives (or positives).

For each grid frame (*a*_*i*_, *b*_*j*_) that is crossing the diagonal, the algorithm calculates a set of 2D peaks, *F *∩ Δ(*a*_*i*_, *b*_*j*_), and checks the peak volume of each. This set consists of all fractal frames that are contained inside (*a*_*i*_, *b*_*j*_). A mental picture is that (*a*_*i*_, *b*_*j*_) must be broken into fractal frames (fractured) to avoid crossing the diagonal. The algorithm searches the subtree Δ(*a*_*i*_, *b*_*j*_) top-down (breadth-first) with a recursive subroutine. A given input frame (*a*, *b*) is split into its father frame *π*(*a*, *b*) and mother frame *μ*(*a*, *b*). Each in turn is then checked as follows: If it is crossing the diagonal, it is further split by giving it recursively as input to the subroutine. If instead it is above the diagonal, it is a fractal frame. With (*a*_*i*_, *b*_*j*_) as input, the subroutine finds *F *∩ Δ(*a*_*i*_, *b*_*j*_). (If instead the input is the root frame (*ρ*_x_, *ρ*_y_), the subroutine will find all fractal frames *F *. This was applied in Algorithm 1.)

**Figure 6:**
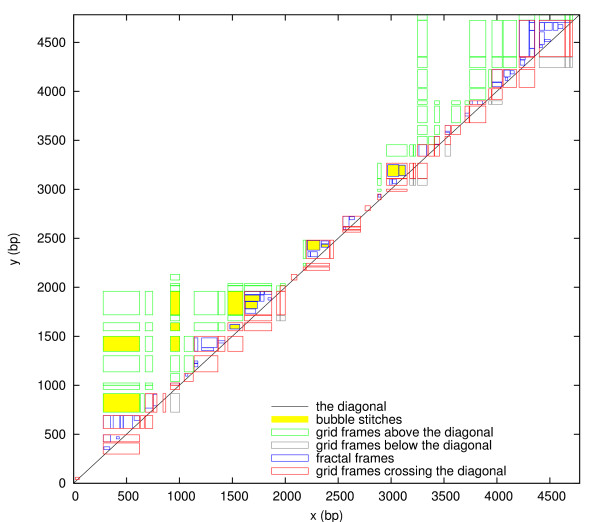
**The footprints of Algorithm 2 plotted in the *xy*-plane**. These are frames that are visited by Algorithm 2 during its search for the bubble stitches (filled yellow). The frames are located in a band along the diagonal, suggesting that the search space is proportional to sequence length. Grid frames below the diagonal (grey) are skipped. Grid frames crossing the diagonal (red) are broken into fractal frames (blue). The bubble stitches (filled yellow) are those grid frames above the diagonal (green) and fractal frames (blue) that have *p*_v_(*a*, *b*) ≥ *p*_*c*_. Frames with side lengths below 20 bp are not shown to unclutter the figure.

Figure 6 shows the resulting search process, by plotting only frames that are processed by the algorithm, while the ignored grid frames are blank. Comparing with Figure 3, we see that the blank areas correspond to the great bulk of grid frames both above and below the diagonal, leaving just an irregular band of frames along the diagonal to be searched. This is a nice geometric illustration of the reduction from *O*(*N*^2^) to *O*(*N *log *N*) in execution time. Figure 6 also shows that some bubble stitches are fractal frames contained inside grid frames that are crossing the diagonal.

The peak volumes *p*_v_(*a*, *b*) of some frames must be calculated. Algorithm 2 spends a considerable fraction of its time on doing these summations. The summation over a bubble frame can be done faster if the frame is big enough, by exploiting the Fixman-Freire approximation *à la *Yeramian [32,41]. This does not improve the time complexity, but significantly reduces the total execution time by some factor.

To compute all helix stitches of the stitch profile, the algorithm follows exactly the same procedure as described above, but in the helix context. Eq. (14b) and the analysis in the previous section applies equally well to the bubble and the helix contexts. The various quantities are, of course, replaced by their helix counterparts. For example, the appropriate diagonal line is applied (Def 6). The main difference is the upper bound on helix peak volume. Since x and y decouples in the helix probability [29],

(19) $$p_{\text{helix}}(x,y)=\frac{p_{\text{helix}}(x,N)p_{\text{helix}}(1,y)}{p_{\text{helix}}(1,N)},$$

we can simply use the peak volume as its own upper bound:

(20) $${\tilde{p}}_{\text{v}}(a,b)=p_{\text{v}}(a,b)=\frac{p_{\text{v}}(a)p_{\text{v}}(b)}{p_{\text{helix}}(1,N)}.$$

The Ξ (*x*, *y*) factor [29] is the counterpart of Ω(*y - x*), but an explicit consideration of its monotonicity is not necessary here, because it is absorbed in the above quantities. A next bigger goat is then calculated and applied in the same way as for bubbles.

## Results and Discussion

### Time complexity

By inspection of Algorithm 2, we observe that it visits at least *O*(*N*) and at most *O*(*N*^2^) matrix elements of *G*. Furthermore, it performs sorting, which is known to scale as *O*(*N *log *N*). The time complexity is therefore between *O*(*N *log *N*) and *O*(*N*^2^). The execution time depends on the fraction of ignored grid frames above the diagonal, which depends on the specific sequence, temperature, and other input parameters. A theoretical analysis of these dependencies is complicated.

Empirical testing of the execution times were done instead, using a test set of 14 biological sequences with lengths selected to be evenly spread on a log scale spanning three decades. A minimum length of 1000 bp was required. Most of the test sequences are genomic sequences, so as to represent the typical usage of the algorithm. The sequence lengths and accession numbers are:

• 1168 bp [GenBank:BC108918]

• 1986 bp [GenBank:BC126294]

• 4781 bp [GenBank:BC039060]

• 7904 bp [GenBank:NC_001526]

• 16571 bp [GenBank:NC_001807]

• 36001 bp [GenBank:AC_000017]

• 48502 bp [GenBank:NC_001416]

• 85779 bp [GenBank:NC_001224]

• 168903 bp [GenBank:NC_000866]

• 235645 bp [GenBank:NC_006273]

• 412348 bp [GenBank:AE001825]

• 816394 bp [GenBank:NC_000912]

• 1138011 bp [GenBank:AE000520]

• 2030921 bp [GenBank:NC_004350]

**Figure 7:**
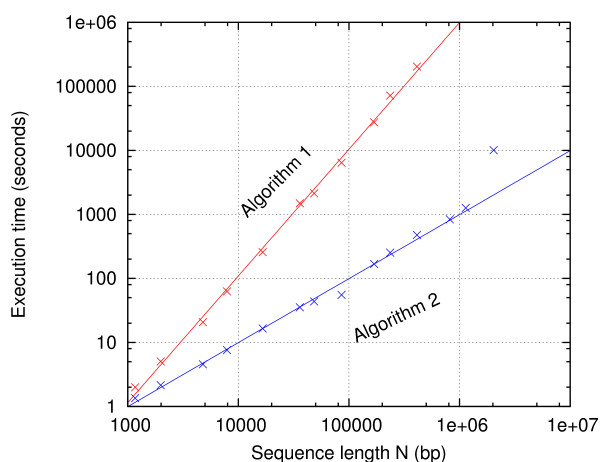
**Algorithm 1 is quadratic and Algorithm 2 is linear**. The log-log plot shows the execution time versus sequence length of Algorithm 1 (red) and Algorithm 2 (blue). The straight lines are fits to the data points with slopes 1.97955 ± 0.02923 (red) and 0.99953 ± 0.02016 (blue).

The algorithms were written in Perl and run on a Pentium 4, 2.4 GHz, 512 KB cache, 1 GB memory, PC with Linux (CentOS). In Figure 7, the speeds of Algorithms 1 and 2 are compared. Algorithm 2 is orders of magnitude faster than Algorithm 1 for sequences longer than 100 kbp. While all the 14 sequences were computed by Algorithm 2, the three longest sequences were aborted by Algorithm 1, because of too long execution times. To ensure that the computational tasks were comparable, all sequences were computed at their melting temperatures *T*_m_, rather than one temperature for all, such that all sequences had the same fractions of helical regions and bubbles. For both algorithms, straight lines were fitted to the data in the log-log plot. For Algorithm 2, however, the longest sequence (2 Mbp) is considered an outlier and thus excluded from the fit. This sequence's execution time was overly increased, because the required memory exceeded the available 1 gigabyte RAM. For Algorithm 1, the slope of the fit is 1.97955 ± 0.02923, suggesting that it has time complexity *O*(*N*^2^). For Algorithm 2, the slope of the fit is 0.99953 ± 0.02016. This is interpreted as the time complexity *O*(*N *log *N*), but with the logarithmic component being too weak to distinguish *O*(*N *log *N*) from *O*(*N*).

**Figure 8:**
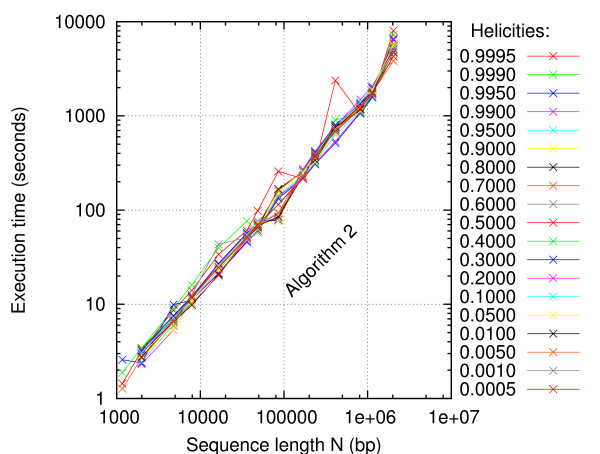
**Algorithm 2 is fast at all temperatures**. The total execution times are plotted versus sequence length for each of the listed helicity values.

The execution time of Algorithm 2 is just as much a property of the underlying energy landscape depending on the input, as it is a property of the algorithm. Could it be that other input parameters and/or sequences than was used in Figure 7 – say, away from the melting points – would exhibit the time complexity *O*(*N*^2^)? Figure 8 shows the speed of Algorithm 2 over the whole melting range of temperatures. Each sequence in the test set was computed at temperatures corresponding to the helicity values: 0.9995, 0.999, 0.995, 0.99, 0.95, 0.9, 0.8, 0.7, ..., 0.2, 0.1, 0.05, 0.01, 0.005, 0.001, and 0.0005. This helicity range approximately corresponds to the temperature range *T*_m _± 10°C and it covers most of the melting transitions. Although the curves for the individual helicity values may not be easily distinguished in Figure 8, it appears that all curves have similar slopes and that they are close to each other, i.e., the variation in execution time is below 50%. This indicates that the helicity (or temperature) value has only a small influence on the total execution time. The time complexity *O*(*N *log *N*) seems to be robust.

**Figure 9:**
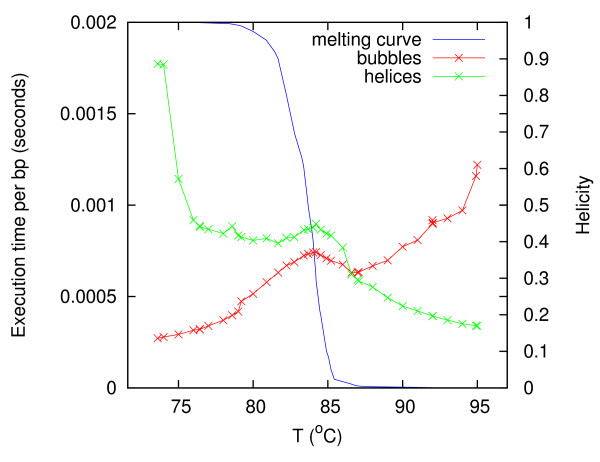
**Bubble and helix execution times versus temperature**. For the sequence [GenBank:NC_001807], the bubble time (red) and helix time (green) divided by sequence length (16571 bp) is plotted versus *T*. The melting curve (blue) shows the helicity Θ (on the right vertical axis) as a function of *T*, indicating the melting midpoint: Θ = 0.5 at *T*_m _= 83.7°C.

However, a stronger temperature dependence is revealed when considering the computations of bubble stitches and helix stitches separately. Two independent subroutines of Algorithm 2 compute the bubble stitches and the helix stitches, both following the procedure outlined in the previous section. The rest of Algorithm 2's computation, including the initial computation of at least four partition function arrays [30], is called the overhead. Correspondingly, the total execution time *t*_total _is the sum of the bubble execution time *t*_bubble_, the helix execution time *t*_helix_, and the overhead execution time *t*_overhead_. By simply switching off the bubble subroutine (i.e. *t*_bubble _= 0) and measuring the total execution time, we obtain *t*_helix _+ *t*_overhead_. Likewise, by switching off the helix subroutine, we measure *t*_bubble _+ *t*_overhead_. In the following, we refer to *t*_bubble _+ *t*_overhead _as the bubble time and *t*_helix _+ *t*_overhead _as the helix time. As an example, Figure 9 shows the results for the 16571 bp [GenBank:NC_001807]. The bubble and helix times are divided by sequence length and plotted as a function of temperature. Both of them have clearly a strong temperature dependence. The melting curve is also plotted in Figure 9, indicating that most of the melting occurs in the temperature range 80–85°C. Plots like Figure 9 were made for each sequence in the test set, but the average behavior is more interesting. To average times of the order *O*(*N*) over sequences of different lengths, one should divide them by sequence length as in Figure 9. However, to plot as a function of temperature would not be meaningful, because the sequences have different *T*_m_'s and different melting ranges. On the horizontal axis, instead, we use a normalized temperature,

(21) $$\tau =\log(\frac{1-\Theta }{\Theta }),$$

defined such that the melting curve becomes a sigmoid:

(22) $$\Theta =\frac{1}{1+\exp(\tau )}.$$

**Figure 10:**
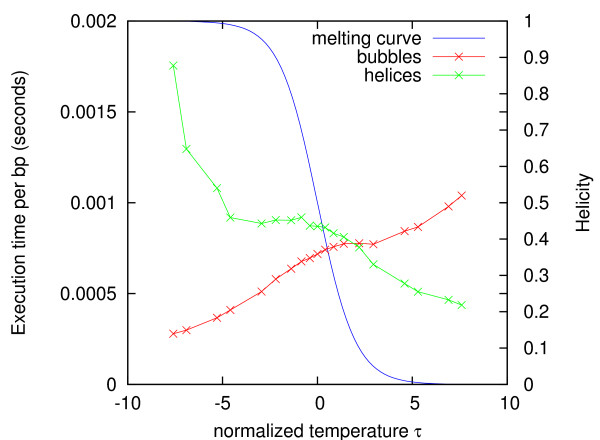
**Sequence-averaged bubble and helix execution times**. The bubble time per basepair (red) and helix time per basepair (green) averaged over all sequences are plotted versus the normalized temperature *τ *for each of the helicity values listed in Figure 8. The melting curve (blue) shows the helicity Θ (on the right vertical axis) as a function of *τ*, indicating the melting midpoint: Θ = 0.5 at *τ *= 0.

For each *τ*-value (or equivalently for each Θ-value), the bubble times and helix times divided by sequence length averaged over all sequences are plotted in Figure 10. The curves have a similar temperature dependence as in Figure 9. The helix time decreases monotonically (except for a shoulder), while the bubble time increases monotonically (except for a shoulder). Both of them have an about four-fold difference between their maximum and minimum. Qualitatively, the curves are kind of mirror symmetric, but the helix time is generally greater than the bubble time, the two curves cross each other at Θ = 0.12. It seems that adding the two curves would give a more or less horizontal curve, i.e., the total execution time has much less temperature dependence.

We may understand this interchange between bubble time and helix time in terms of the melting process. If we assume that the bubble time is proportional to the area of the footprint in Figure 6, and that this is proportional to the average length of potential bubbles at that temperature, then we would expect the bubble time to increase with temperature, because bubbles grow as DNA melts. Likewise, we would expect the helix time to decrease with temperature, because helical regions diminish as DNA melts.

In this article, Blake & Delcourt's parameter set [42] as modified by Blossey & Carlon [43] was used with [Na^+^] = 0.075 M. Parameters for the loop entropy approximation was obtained with our online tool [31,44]. The maximum depth and probability cutoff parameters were *D*_max _= 5 and *p*_c _= 0.01 in Figs. 3, 4, 5, 6, *D*_max _= 3 and *p*_c _= 0.02 in Figure 7, and *D*_max _= 3 and *p*_c _= 0.0001 in Figs. 8, 9, 10. The sequence [GenBank:BC039060] was used for producing Figs. 2, 3, 4, 5, 6. A systematic test of how the execution time depends on *D*_max _and *p*_c _has not been performed.

### Discussion

For an algorithm to be called efficient, it should solve the task at hand with optimal time complexity. It should not introduce approximations, that would just amount to a reformulation of a simpler, but different task. In this study, the task is to compute a stitch profile based on the Poland-Scheraga model with Fixman-Freire loop entropies. With this model, the time complexity must be at least *O*(*N *log *N*). Indeed, this is achieved by Algorithm 2 under a wide range of conditions. Algorithm 2 does not acquire a speedup by any *windowing *approximation, by which the sequence would first be split into smaller independent sequences. Neither does it rely on limiting the problem to a maximal bubble length. Therefore, Algorithm 2 is efficient. In computational RNA and protein studies, a maximal loop size is sometimes imposed as a heuristic for reducing time complexity by one order. Similarly, a maximal DNA bubble size of 50 bp has been reported in computations of low temperature bubble probabilities in the Peyrard-Bishop-Dauxois model [14]. In contrast, Algorithm 2 can find bubbles of whatever size at any temperature. In the 48502 bp [GenBank:NC_001416], for example, bubbles and helical regions may be up to around 20000 bp long [45]. Although Algorithm 2 has no explicit notion of a maximal bubble length, it may implicitly detect length limitations for both bubbles and helical regions by the absence of the "next bigger goat". In this way, Algorithm 2 can adapt to the input sequence. This adaptation is evident in Figure 10, where the bubble execution time grows as bubbles get bigger at higher temperatures. Conversely, the helix execution time decreases as the helical regions gradually melt away.

However, the time complexity was not proven to be *O*(*N *log *N*) under all conditions. It is still an open question whether there is a transition to time complexity *O*(*N*^2^) in some peripheral regions of the input parameter space. But based on results so far, a fast computation would be expected in most situations.

How fast is Algorithm 2? Figures 7 and 8 show that the Perl implementation runs on an old desktop PC at the speed of roughly 1000 basepairs per second. With today's computers, assuming twice that speed and enough memory, the *E. coli *genome would take 39 minutes, the yeast genome would take 1.7 hours, and the largest human chromosome would take 35 hours. In some types of low temperature melting studies, the features of interest are the bubbles rather than the helical regions. In such applications, switching off the computation of helix stitches can speed up the algorithm several times. As Figure 10 indicates, the helix time is about twice the bubble time at helicity equal to 0.95, that is, the speedup would be about threefold. The largest human chromosome would be done in ten hours. On a computer cluster, the human genome could be computed in a day. Such bubbles could then be compared to TFBS, TSS, replication origins, viral integration sites, etc.

The required memory grows with sequence length and for sequences longer than 2 Mbp, more than 1 GB was needed. The memory usage has not been tested further and the space complexity has not been discussed in this article. Some memory optimization of the Perl implementation must be done before such test can reflect the space complexity. While the algorithm is efficient in terms of time complexity, the code has room for optimization of both speed and memory usage. However, the space complexity is believed to be *O*(*N*), which means that the algorithm would eventually become out of memory for long enough sequences. A standard solution is to introduce efficient use of disk space instead, which could reduce the memory usage to *O*(1), without increasing the time complexity.

The fast algorithm presented in this paper relies on a factorization of certain upper bounds, which in turn relies on a factorization of partition functions in the Poland-Scheraga model, see Eq. (16). In general, it seems that a factorization of partition functions is essential for solving DNA and RNA models in polynomial time. However, some DNA melting models that explicitly consider supercoiling do not allow such a factorization [46]. For the purpose of genomic applications, supercoiling and a number of other *in vivo *interactions and constraints in the cell should ideally be accounted for in future DNA model development [1,15]. Such modelling requires quantitative knowledge yet to be obtained experimentally. When such data becomes available, a main challenge will be to develop models and algorithms that can be solved in time *O*(*N *log *N*), which is necessary for many genomic applications.

## Conclusion

The fast algorithm described in this article enables the computation of stitch profiles of genomic sequences. Melting features of interest, such as bubbles, helical regions, and their boundaries, are computed directly, rather than relying on visualization or educated guesses. The algorithm is exact. It does not achieve its speed by approximations, such as windowing or maximal bubble sizes. Genomewide comparisons of bubbles with TSS, replication origins, viral integration sites, etc., are proposed. The algorithm is available in Perl code from the author. Online computation of stitch profiles is available on our web server, which has recently been upgraded to run Algorithm 2 [31,44].

## Competing interests

The author declares that they have no competing interests.
